# Unveiling the Metabolic Maze: FDG PET/CT Findings in Peritoneal Carcinomatosis - A Case Series

**DOI:** 10.22038/AOJNMB.2024.78270.1552

**Published:** 2024

**Authors:** Vijay Singh, Dinesh Srivastava, Neha Kotarya, Manish Ora, Prasanta Kumar Pradhan

**Affiliations:** Department of Nuclear Medicine Sanjay Gandhi Post Graduate Institute of Medical Sciences Lucknow, India

## Abstract

Peritoneal carcinomatosis (PC), the spread of cancer cells in the peritoneum, is a significant concern in advanced gastrointestinal and gynecological cancers. This case series includes findings on the appearance and pattern of PC on ^18^F-fluorodeoxyglucose positron emission tomography/CT (^18^F-FDG PET/CT). The primary sources of peritoneal dissemination are direct invasion from abdominal or pelvic tumors and metastatic spread from distant tumors. The accurate preoperative diagnosis and quantification of PC play a vital role in determining the appropriate treatment approach, with a particular emphasis on surgical planning. Several imaging modalities have been employed in preoperative evaluation, such as computed tomography (CT), magnetic resonance imaging (MRI), and ^18^F-FDG PET/CT. Among these modalities, ^18^F-FDG PET/CT has demonstrated improved anatomical localization and accurate information about the nature of pathological findings. The case series showcases four cases that illustrate the imaging characteristics of PC on FDG PET/CT. FDG PET/CT plays a vital role in diagnosing and assessing PC, aiding in its detection, staging, and treatment planning. It surpasses conventional imaging techniques in identifying and characterizing lesions and detecting the primary tumor site in cases where its location is unknown. Furthermore, FDG PET/CT additionally assists in evaluating treatment response and monitoring disease progression, providing insights into treatment effectiveness and guiding patient management decisions.

## Introduction

 Peritoneal seeding is a well-known dissemination route in advanced gastro-intestinal and gynecological cancers but may also occur in primary peritoneal tumors. The term "peritoneal carcinomatosis" was created by Sampson et al. (1) to characterize the spread of ovarian cancers that involve the peritoneal serous membrane. The most common form of intraperitoneal dissemination is secondary. It occurs by direct invasion through the gastrointestinal (GI) wall or ovarian capsule in advanced GI or ovarian tumors. Another route is metastatic spread from advanced systemic tumors such as breast and lung (2, 3). The mesothelial layer of the serosa is the source of rare primary peritoneal mesothelioma. 

 Iatrogenic dissemination is the most common mechanism of peritoneal spread from abdominal tumors (4). Pseudomyxoma peritonei is abdominal cavity dissemination from a low-grade myxoid tumor of the appendix or ovarian capsule (4). The peritoneal carcinomatosis's extent and volume are significant prognostic markers. Preoperative diagnosis and quantification of PC are crucial for management, particularly in the surgical approach. Surgical exploration is the gold standard (5). Multiple imaging modalities have been employed for the preoperative PC assessment, including CT, MRI, and ^18^F-FDG PET/CT (6). ^18^F-FDG PET/CT, with contrast, has an emerging role in cancer management. The use of ^18^F-FDG PET/CT enables better anatomical localization of intra- and extra-pelvic structures, and their relationship with ^18^F-FDG uptake may offer more accurate information about the nature of the pathological findings (7). Here, we describe the imaging feature and pattern of PC on FDG PET/CT.

## Case 1

**Figure 1:**
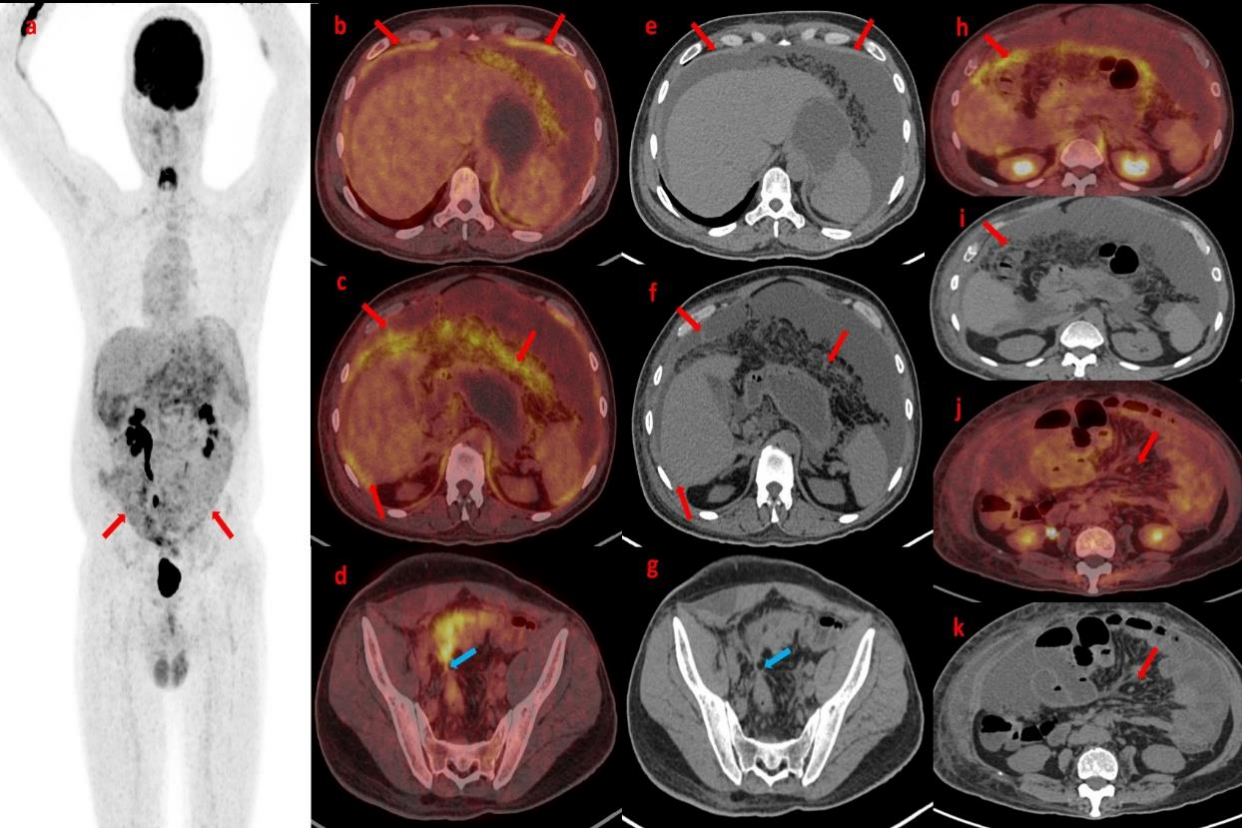
Maximum Intensity Projection (MIP) image (**a**) shows physiological tracer uptake in the brain, kidney, and urinary bladder. Abnormal FDG uptake is observed in the entire peritoneum with nodules (**red arrow**). Axial section Fused PET/CT images (**b**,** c**) and axial CT images (**e**, **f**; **red arrow**) show diffuse thickening of the peritoneum, omental caking with increased FDG uptake and gross ascites. Axial section Fused PET/CT (**d**; **blue arrow**) and axial CT (**g**; **blue arrow**) from the pelvis indicate a narrowing of the sigmoid colon with increased uptake proximally. Postoperative histopathology confirmed Ca Sigmoid colon. Further axial PET/CT images (**h**, **j**) and corresponding Axial CT images (**i**, **k**) reveal mesenteric stranding with nodules and increased FDG uptake. Both sections also show FDG avid ascites; all these findings are PC characteristics

 A 52-year-old male presented with a six-month history of abdominal pain, loss of appetite, and weight loss; the pain was spasmodic, mild to moderate, and recurrent. It was associated with recurrent constipation and abdominal distention, which improved after passing stools. The patient had a low-grade fever (up to 100°F) for one month, which responded to antipyretics. On examination, the abdomen was soft-distended, with mild tenderness and palpable thickening in the right hypochondrium. An abdominal ultrasound revealed ascites. Fluid analysis revealed a low Serum Ascites Albumin Gradient (SAAG). A CT enterography showed gross ascites with peritoneal thickening, diffuse omental nodularity, and caking. The colonoscope could not be advanced beyond the sigmoid colon. An ultrasound-guided biopsy from the omentum revealed metastatic adenocarcinoma. Tumor cells were positive for cytokeratin CK20, CDX2, and SATB2 and negative for CK7 and Ttf 1. ^18^F-FDG PET/CT with contrast revealed gross ascites with metabolically active asymmetrical sigmoid colon thickening. Diffuse peritoneal thickening with extensive nodular omental stranding was also noted (Figure 1). Few metabolically active serosal deposits were seen in the jejunal-ileal loop. Metabolically active right supraclavicular, right internal mammary, and multiple abdominal-retroperitoneal lymph nodes were also noted. The overall feature suggested a sigmoid colon primary with PC. The patient received two cycles of Oxaliplatin and Capecitabine-based chemotherapy. After two cycles, he was lost to follow-up.

***Case 2***

 The 42-year-old woman presented with abdominal distension two years ago. An abdominal ultrasound revealed ascites and a pelvic mass. A large solid cystic mass with papillary excrescence was identified on trans-vaginal ultrasound. Biochemical examination showed elevated CA-125 (>200 unit/ml) levels. 

 The patient underwent a total abdominal hysterectomy with bilateral salpingo-oophorectomy (TAH with BSO). 

 Histopathology (HPE) revealed Epithelial Ovarian cancer; she received six cycles of chemotherapy. The patient remained asymptomatic for two years until she developed abdominal distension. An ultrasound revealed disease recurrence, and she was started on paclitaxel and carboplatin-based chemo-therapy. However, she did not respond to the regimen and developed recurrent vomiting. 

 Biochemical parameters showed mildly deranged liver function tests and raised total leukocyte counts (~25,000/µl). She reported fatigue and progressive weight loss.

**Figure 2:**
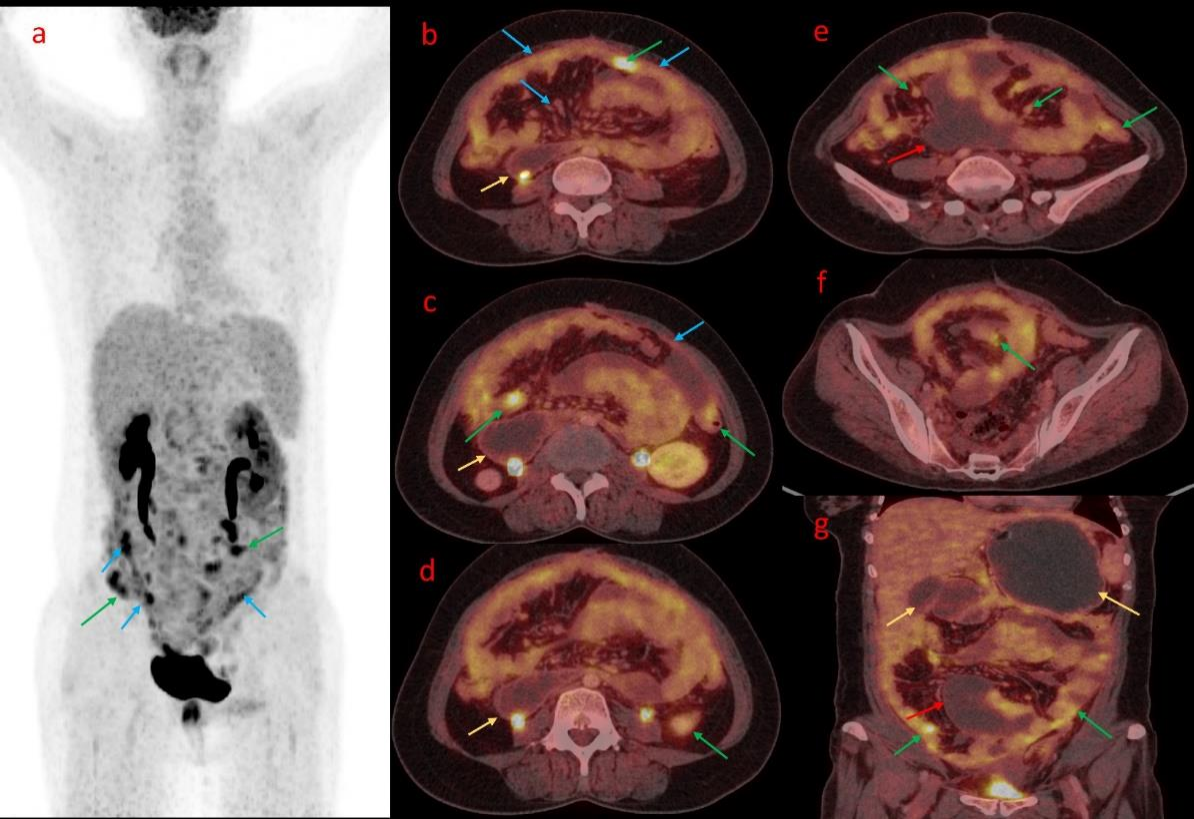
FDG PET/CT Maximum Intensity Projection Images (**a**) in a Post-op Case of Ca ovary reveals increased FDG uptake in the peritoneum with multiple nodularities (**Blue and green arrow**). Fused Axial PET/CT images (**b**,**c**) revealed diffuse peritoneal thickening with increased FDG uptake (**blue arrow**); peritoneal and serosal deposits (**green arrow**) in and dilated duodenal loops were also noted (**yellow arrow**). Further Axial fused PET/CT images (**d**, **e**, **f**) revealed multiple peritoneal deposits (**green arrow**), mesenteric nodularity (**blue arrow**), intraperitoneal fluid collection (**red arrow**), and dilated duodenal loop. Coronal fused PET/CT image (**g**) revealed FDG avid peritoneal thickening, mesenteric nodularity, Serosal deposits, intraperitoneal fluid collection, and dilated bowel loops (**yellow**) suggestive of peritoneal carcinomatosis

 On physical examination, the patient appeared pale and fatigued. Abdominal distension and tenderness were noted. There were no palpable lymph nodes in the cervical, axillary, or inguinal regions. She was referred for PET/CT to determine the extent of the disease. The 18FDG PET/CT with contrast showed ascites with FDG avid variable sizes peritoneal, mesenteric, and serosal soft tissue deposits (Figure 2). The overall features were suggestive of PC. The patient deteriorated rapidly and passed away in a few weeks.

***Case 3***

 A 57-year-old female presented with a history of constipation for one year; she had recurrent abdominal distention for one month and underwent paracentesis. An abdominal ultrasound revealed Chronic Liver Disease. Ascitic fluid showed low SAAG ascites. Serum creatinine was elevated, and a diagnosis of acute kidney disease was made. Ascitic fluid was positive for malignant epithelial cells (adenocarcinoma). The patient was referred for ^18^F-FDG PET/CT with contrast to investigate the carcinoma of unknown primary. It revealed a solid cystic lesion in the left adnexa with FDG uptake in the solid component. In addition, FDG uptake was also noted in the endometrial cavity. 

**Figure 3:**
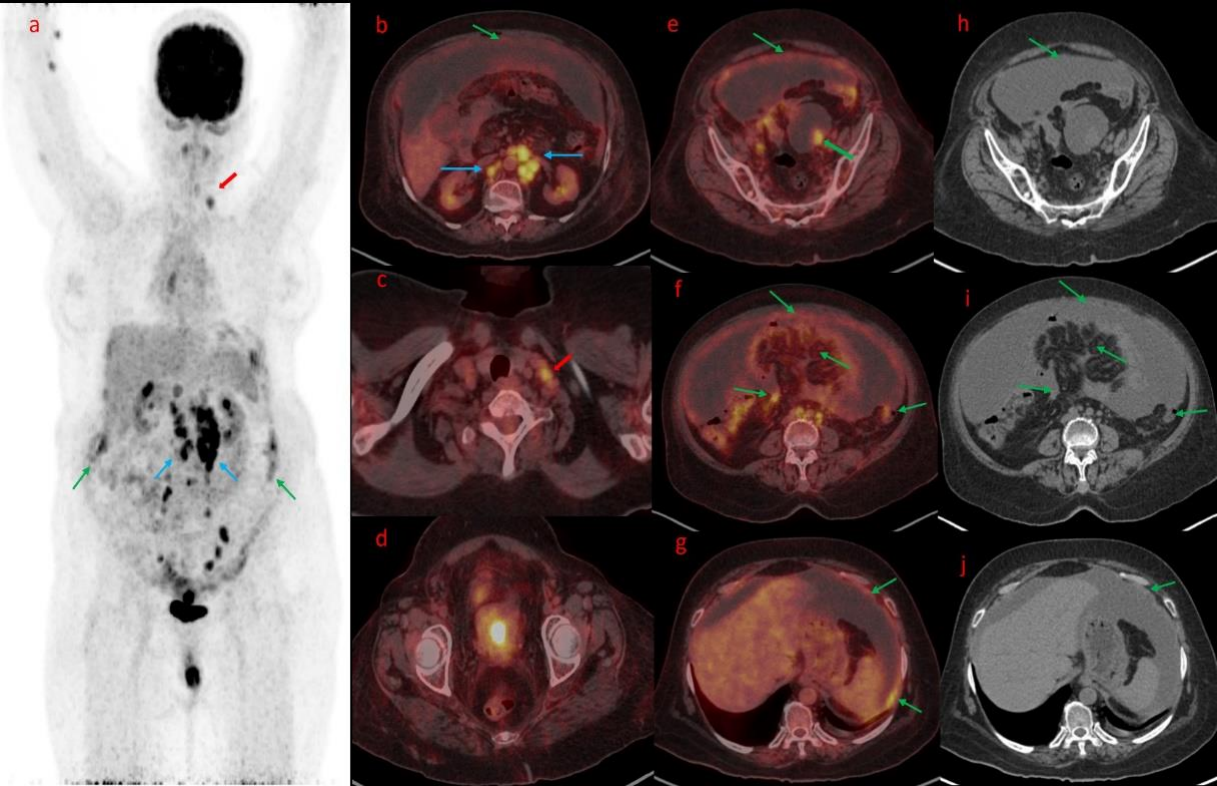
Maximum Intensity Projection image (**a**) revealed increased FDG uptake in the peritoneum (**Green arrow**) with nodularity and multiple FDG avid abdominal nodes (**blue arrow**) and a left supraclavicular lymph node (**Red arrow**). Axial section Fused FDG PET/CT (**b**, **c**) revealed gross ascites with FDG avid peritoneal thickening (green arrow), Multiple FDG avid abdominal nodes (**blue arrow**), and left supraclavicular lymph node (**red arrow**). Axial Fused PET/CT (**d**) from the pelvis revealed increased FDG uptake in the endometrium cavity. Axial Fused FDG PET/CT images (**e**, **f**, **g**) and corresponding CT (**h**, **i**, **j**) revealed diffuse peritoneal thickening with peritoneal and serosal deposits, omental thickening with increased FDG uptake (**green arrow**), and ascites. Fused Axial PET/CT (**e**) and corresponding CT images (**h**) revealed a solid cystic lesion in the bilateral adnexal region with increased FDG uptake in the solid component of the left adnexa (**e**, **green arrow**)

 Multiple abdominal, retroperitoneal, mediastinal, internal mammary, and supraclavicular lymph nodes were also noted. The PET/CT also revealed gross ascites with metabolically active diffuse peritoneal, omental thickening, and soft tissue stranding. These imaging findings suggested a primary ovarian malignancy with PC (Figure 3). The CA-125 tumor marker level was elevated (96.26 u/ml). The patient was being considered for a Paclitaxel and bevacizumab-based chemotherapy regimen and is currently receiving chemotherapy.

***Case 4***

 A female aged 45 years presented with abdominal pain for one year; she had no menstrual irregularities, weight loss, fever, or other systemic symptoms. On examination, she was pale with an enlarged abdomen exhibiting shifting dullness. She had elevated serum CA-125 level (250 U/mL, normal range <35 U/mL). 

 The abdominal ultrasound revealed a voluminous uterus with a thickened endometrium and a right hemorrhagic ovarian cyst measuring 8×7 cm. The left adnexa were unremarkable, with no ascites or lymphadenopathy. The patient underwent abdominal contrast-enhanced computed tomography (CECT). It showed bilateral enlarged adnexal solid-cystic masses with areas of calcifications indicative of malignant ovarian neoplasms. Ultrasound-guided fine needle aspiration cytology (FNAC) of the right ovarian mass suggested an ovarian epithelial neoplasm. 

**Figure 4:**
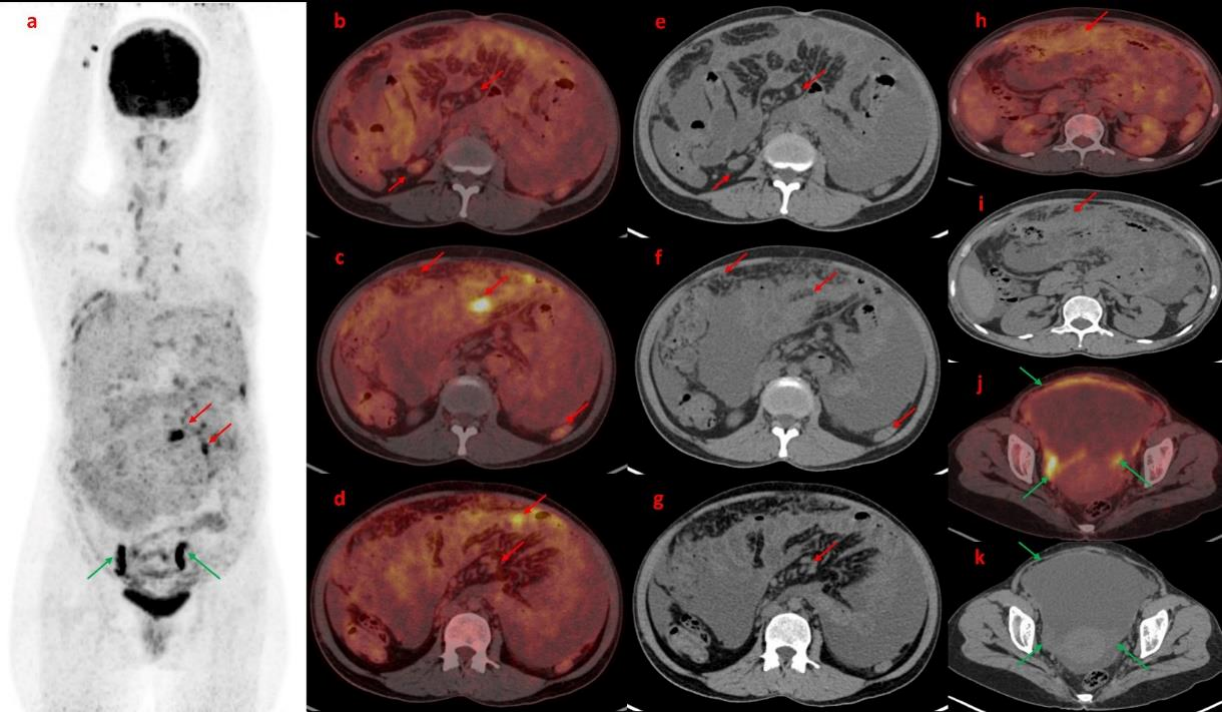
Maximum Intensity Projection (**a**) image revealed diffuse increased uptake in the peritoneum with few FDG avid nodules and increased FDG uptake in the bilateral adnexal region. (**Green arrow**). Axial section Fused FDG PET/CT images (**b**, **c**, **d**) and corresponding axial CT images (**e**, **f**, **g**) revealed omental thickening, omental nodules, few peritoneal deposits, and mesenteric thickening with nodularity. Ascites can also be noted in all these sections. Further Fused Axial PET/Ct images (**h**, **j**) and axial CT images (**I**, **k**) revealed omental caking and peritoneal thickening with increased FDG uptake (red arrow) and ascites. A fused axial PET/CT image (**j**) and corresponding axial CT image (**k**) also revealed an FDG avid bilateral adnexal lesion with pelvic ascites

^18^F-FDG PET/CT with contrast revealed a metabolically active bilateral adnexal mass. Extensive lymphadenopathy, gross ascites, and peritoneal and omental thickening were noted as indicative of PC (Figure 4). The patient underwent three cycles of palliative chemotherapy (carboplatin and paclitaxel). She had a partial treatment response with improvement in symptoms. She is receiving chemotherapy.

## Discussion

***Peritoneum and malignant involvement***

 The peritoneum and peritoneal cavity are essential in intestinal motility and lymphatic circulation. 

 Peritoneal dissemination refers to the proliferation of cancer cells in the peritoneum and peritoneal cavity. Peritoneal involvement may be seen as secondary metastasis from a distant advanced tumor. Direct invasion from abdominal or pelvic tumors is also noted, notably in gastric, colon, or ovarian cancer. Tumors breach the gastrointestinal lining or ovarian capsule (2). 

 Previous studies have emphasized the possible role of diagnostic biopsy and surgical procedures in peritoneal dissemination. After tumor resection, peritoneal fluid cytology has frequently revealed malignant cells in gastric cancer (8). Peritoneal spread may also occur through postoperative leakage from transect lymphatic channels. After implantation of the tumor cell on the peritoneum, the tumor progression depends on numerous factors. The phenomenon of metastatic inefficiency refers to the ineffectiveness of cancer cells in spreading through the vascular or lymphatic channels. 

 Weiss (9) demonstrated that the vascular space resists tumor cell implantation. Unfortunately, peritoneal cavity cancer implantation has no effective defense. Peritoneal implantation leads to inflammation. Inadequate tumor cells killed by inflammatory cells result in invasion (10). A fibrin layer shields cancer cells from the body's immune system. In addition, trapping wound-healing factors in the peritoneal cavity stimulates the growth and de-differentiation of cancer cells (11). 

 Tumoral involvement of the peritoneum leads to dysfunctions and results in ascites. The extent of peritoneal involvement, organ invasion, and tumor biology, such as the type and grade of the tumor, determines the severity of the symptoms (2). Cancers with moderate and high grades have spread early, even in the presence of significant ascites, due to the expression of adhesion molecules in the cancer cell. Another pattern is noted in low-grade tumor cells; their low biological aggressiveness prevents them from adhering to the peritoneal surface near the primary tumors. Moreover, the high mucin production causes a complete redistribution in the peritoneal cavity. Finally, invasive mucinous tumors that produce large amounts of mucus and impair cell adhesion are characterized by widespread cancer dissemination (12).

***Multimodality Imaging to evaluate Peritoneal Carcinomatosis***

 Multiple imaging modalities such as ultrasonography (USG), CT, MRI, and PET/CT are utilized in PC. (6, 13, 14). USG is the initial diagnostic modality for patients with suspected PC. It detects peritoneal fluid along with peritoneal implants. Nevertheless, it is imperative to understand the dependency of the operator's expertise (6). Although the USG is a non-radiation imaging modality, the sensitivity in detecting PC is variable (6, 14). CT scan is a widely utilized cross-section imaging modality for assessing PC (6, 13, 14). The imaging includes administering intravenous, oral, and rectal contrast agents to attain favorable imaging. (6, 15). The detection accuracy of CT in PC is variable depending upon the location of the lesion. The gutters and free surfaces of the spleen and liver exhibit the highest detection rates, whereas the pelvis and mid abdomen display comparatively lower detection rates. 

 The detection rates of tumor nodules are also influenced by the size, with smaller lesions (<0.5 cm) exhibiting lower sensitivity compared to larger nodules (>5 cm) (15). The ability of CT scans to identify lesions is affected by the contouring of intraabdominal structures. In general, nodular lesions with well-defined volumes are reliably diagnosed with CT if their size exceeds the spatial resolution of the CT scan (6).

 Notably, de Bree et al. (16) have observed a significant variability (60% to 76%) among radiologists when interpreting CT scans of PC originating from colorectal sources. Small tumor nodules (less than 1 centimeter) have a detection rate that ranges from 9% to 24%. 

 Furthermore, detecting lesions on the small bowel and its mesentery, crucial for determining the need for surgical intervention, is also subject to variability (6, 13-15). 

 MRI provides an enhanced visualization of soft tissues. Additionally, it permits the assessment of the parietal or visceral peritoneum in the presence of ascites (17). The overall sensitivity of contrast-enhanced MRI and CT are comparable (17, 18), although MRI may possess a potential advantage (19). MRI acquisition does not involve radiation and takes a longer time. Standard MRI protocol includes T1-weighted, T2-weighted, fat-suppressed, dynamic contrast-enhanced, and diffusion-weighted imaging (DWI) (18, 20). The DWI has demonstrated its efficacy in staging, differential diagnosis, and assessment of treatment response. Nevertheless, respiratory and cardiac motion artifacts can impede the visualization of lesions below the diaphragm and within the hepatic subcapsular region (18, 20). MRI may possess certain limitations to accurately detect bowel wall involvement, necessitating experienced radiologists' expertise for accurate interpretation (21). 

***Functional FDG PET/CT Imaging in PC***

 PET/CT utilizes the glucose analog radiotracer ^18^F-FDG that concentrates on the regions of high metabolism, such as tumors, inflammatory lesions, and physiologically active tissues. The FDG PET/CT imaging technique incorporates functional and anatomical characteristics, offering significant insights into the extent of disease involvement. The integrated use of PET and CT images facilitates accurate localization and characterization of lesions, aiding in diagnosing, staging, and managing patients. PC exhibits diverse manifestations on FDG PET/CT imaging (22). It may be seen as focal or diffuse aberrant uptake in the bowel serosa, omental fat, and peritoneum (18). The nodular or diffuse uptake, along with peritoneal serous membrane thickening, suggests the presence of tumor cells infiltrating the peritoneal serous membrane (22, 23). Tumoral proliferation can occur through the displacement of omental fat by a solid mass or the formation of small nodules within the adipose tissue (22, 23). PC is prominently noted in the paracolic gutters, the pouch of Douglas, the sigmoid mesocolon, the ileocecal junction, and the anterior parietal peritoneum. The presence of visceral peritoneum involvement has the potential to result in liver surface scalloping (22, 23), a phenomenon that can resemble hepatic metastases. Ascites, in the absence of FDG uptake, indicate the existence of fluid that is either free or confined within specific areas in the peritoneal cavity.

 Nevertheless, the metabolic activity of peritoneal implants within the ascites can significantly increase FDG uptake. Mesenteric invasion and fixation accompanied by FDG uptake indicate the potential disease involvement. These manifestations may lead to anomalous fixation of the small intestine, thickening of the gastric walls, increased FDG uptake in mesenteric fat, and the existence of stellate mesenteric masses or coalesced mesenteric nodules (22, 23).

 A meta-analysis (14 studies, 671 patients) accessed the diagnostic accuracy of ^18^F-FDG PET/CT in PC across different cancers yielded a high diagnostic accuracy (ROC-0.92) (7). The ^18^F-FDG PET/CT yielded a sensitivity of 0.87 [95% CI (0.77-0.93)] and a specificity of 0.92 [95% CI; (0.89-0.94)] (7). Panagiotidis et al. (24) reported a sensitivity, specificity, positive predictive value, negative predictive value, and accuracy of 92.4%, 85%, 94.2%, 81%, and 91%, respectively, the per-patient basis for detecting PC (24). Similar results were obtained by a retrospective analysis conducted by Kim et al. in detecting PC in ovarian cancer patients (25).

 Nevertheless, certain studies have documented lower sensitivity of ^18^F-FDG PET/CT in detecting PC. Lopez-Lopez et al. compared ^18^F-FDG PET/CT with CT in the preoperative staging of primary or recurrent ovarian cancer, who was candidates for cytoreductive surgery and hyperthermic intraoperative intraperitoneal chemotherapy. 

 The authors reported the sensitivity, specificity, positive, and negative predictive values of ^18^F-FDG PET/CT for PC as 24%, 93%, 66%, and 68%, respectively (26). PET/CT has low accuracy for detecting serosal implants mimicking physiological bowel activity, low metabolic activities in mucinous pathology, and small implants below the spatial resolution of the scanner. On the other hand, PET/CT may be able to detect nodal metastases in non-enlarged lymph nodes. PET/CT has a 72-100% sensitivity and a 40-90% specificity for detecting clinically and biochemically silent relapse (6, 14, 18). 

 Although some studies have demonstrated that the addition of PET/CT to routine diagnostic imaging can affect staging in 54-64% of cases and influence clinical decisions in 34-59% of cases (18), the findings are inconsistent, and there is ongoing debate regarding its value compared to standard imaging prior to cytoreductive surgery (26).

 The presented case series highlights the usefulness of FDG PET/CT for assessing individuals with suspected PC. This imaging modality offers significant insights into the extent and metabolic activity of the disease, thereby aiding in its diagnosis, staging, and planning of treatment strategies. Precise localization and comprehensive characterization of peritoneal lesions, assessment of lymph node involvement, and detection of distant metastases are imperative to establish suitable approaches for managing the condition. 

 Moreover, the utilization of FDG PET/CT has demonstrated its beneficial role in detecting the primary tumor site in cases where its location is uncertain, as exemplified in Cases 2 and 3. The PET/CT findings in all four cases within this series consistently demonstrated indications of PC, a frequently observed pattern of metastasis in ovarian, colorectal, and gastrointestinal malignancies. The significance of early detection and accurate staging of PC cannot be overstated, considering its correlation with unfavorable prognosis and the limited therapeutic interventions available.

 The advantage of FDG PET/CT lies in its ability to detect increased metabolic activity exhibited by malignant cells. The uptake of FDG by neoplastic reflects their increased glucose metabolism, a characteristic feature of malignancy. Integrating metabolic information obtained from positron emission tomography (PET) with anatomical imaging derived from CT facilitates enhanced accuracy in identifying and characterizing lesions, surpassing the capabilities of conventional imaging techniques alone. In addition to its diagnostic and staging capabilities, FDG PET/CT plays a crucial role in evaluating treatment response and monitoring the progression of diseases. The analysis of alterations in FDG uptake after therapy provides significant insights into the effectiveness of treatment, guiding subsequent decisions regarding patient management. 

 Nevertheless, it is imperative to acknowledge that the FDG PET/CT results must be assessed alongside clinical and histopathological information to ensure precise diagnosis and effective treatment planning.

***Treatment in PC***

 The treatment landscape for PC has evolved significantly, challenging the traditional notion of it being a terminal condition. Cytoreductive surgery (CRS) plus intraperitoneal chemo-therapy is now thought to be the best course of treatment. The advantages of CRS with hyper thermic intraperitoneal chemo-therapy (HIPEC) or Early Postoperative Intraperitoneal Chemotherapy (EPIC) are highlighted for patients with colorectal cancer; however, it is also utilized in gastric cancer, ovarian cancer, neuroendocrine tumors, peritoneal meso-thelioma, and primary peritoneal carcinoma. Cytoreductive surgery procedure involves pelvic peritonectomy, greater omentectomy, lesser omentectomy, cholecystectomy, multiple resections of the bowel, diaphragmatic stripping, and removal of the liver capsule, sigmoid, rectum, mesorectum, female genital tract and splenectomy in cases of involvement (14, 27).

 One established cytoreductive surgical approach involves using a no-touch isolation technique (NTIT) during primary tumor surgery. This technique completely removes adjacent invaded structures and surgical margins deep in healthy tissue to reduce the incidence of peritoneal metastasis. However, recent clinical trials have raised questions about the superiority of NTIT over conventional techniques, emphasizing the need for further treatments (28).

 Intraperitoneal chemotherapy has gained widespread use compared to systemic chemotherapy, as not all systemically applied chemotherapy agents can efficiently reach the peritoneum due to the peritoneum-plasma barrier (29). HIPEC utilizes specific chemicals and elevated temperatures to eliminate tumor cells, primarily studied in PC related to colorectal, mucinous appendicular adeno-carcinoma, and ovarian cancer (5, 14, 27, 30–33). Its key benefits include maintaining a concentrated regional reagent level, with blood draining via the portal vein to the liver, potentially suppressing liver metastasis. 

 Additionally, 41–43°C hyperthermia directly combats tumor cells by inhibiting RNA synthesis, inducing mitotic arrest, increasing lysosome numbers and activity, enhancing cytotoxicity of certain chemotherapy drugs, and improving tissue penetration (34). Clinical studies evaluating factors influencing outcomes of CRS-HIPEC have suggested that complete procedures positively impact patient survival. 

 Repeated CRS-HIPECs appear beneficial for patients with limited peritoneal metastasis, indicating its potential in specific cases (35, 36).

 A novel technique known as pressurized intraperitoneal aerosol chemotherapy (PIPAC) has been explored for clinical applications. In comparison to systemic chemotherapy and conventional peritoneal cavity administrations, PIPAC offers advantages such as optimizing chemical concentrations uniformly, enhancing drug penetration through increased intraperitoneal pressure, restricting blood outflow, and adjusting peritoneal cavity conditions for improved tissue targeting (5, 27, 37).In a cohort of 26 patients focusing on diffuse malignant peritoneal mesothelioma (DMPM), patients who underwent resection after PIPAC had significantly better median progression-free survival (33.5 vs. 7.4 months) (5,27,38). 

 PIPAC, especially when repeated, has shown promise in making previously unresectable diseases amenable to secondary treatments like cytoreductive surgery (CRS) with HIPEC (39).

 Electrostatic precipitation PIPAC (ePIPAC) and hyperthermic PIPAC (hPIPAC) are emerging methods for improved penetration and distribution. ePIPAC employs electrostatic precipitation of aerosols, and hPIPAC involves applying cisplatin at elevated temperatures (38.8-40.2 ⁰C) (40,41). These innovative methods, including PIPAC variants, offer potential solutions for treating unresectable peritoneal metastases and require additional exploration for their feasibility and effectiveness (42–44). High-intensity ultra-sound (HIUS) has also been studied to treat several solid tumors, and it serves the purpose of improving tissue penetration, showing measurable microscopic changes on the peritoneal surface with minimal damage (5).

 Neoadjuvant Intraperitoneal and Systemic Chemotherapy (NIPS) is a novel method to enhance access to CRS, particularly for cases where tumor features are unsuitable for direct surgery. A meta-analysis of eight retrospective studies involving 373 patients with peritoneal metastasis from gastric cancer revealed that NIPS combined with surgery significantly improved survival compared to those without surgery (5,45), increasing the likelihood of achieving R0 resection (45). While promising, additional clinical trials and research are needed to confirm and evaluate this hypothesis.

 Some drugs and immunotherapies can help prevent peritoneal metastasis. Zang et al. suggested LPPR4 as a new target, as it enhances peritoneal metastasis of gastric cancer (46). 

 CXCL12-CXCR4/CXCR7 signaling protects tumor cells from apoptosis, induces EMT, and changes cell adhesion molecules. AMD3100 (Plerixafor) is a CXCR4 blocker tested for gastrointestinal tumors (47). PD-L1 expression is high in peritoneal metastasis. CMP-001 is a virus-like particle that activates immune cells and interferon-alpha (5, 48–50). Oncolytic virotherapy uses viruses to deliver different agents, such as genes, toxins, radiotherapy, and immunomodulators. JX-594 is an oncolytic virus that stimulates immune cells and can work with checkpoint inhibitors to kill metastases (5, 27, 51). Localized chemotherapy, exemplified by methods like HIPEC and PIPAC, can potentially reduce systemic drug toxicity and maintain higher concentrations in specific areas.

 New delivery systems, including biocompatible carriers like hydrogels, cells, and peptides, are under investigation for treating peritoneal metastasis. Hydrogels, three-dimensional networks of crosslinked hydrophilic polymer chains, can be designed to respond to pH, temperature, and physical stimuli, safeguarding contents and delivering them selectively (52). Various hydrogel-based delivery systems have demonstrated feasibility in peritoneal metastasis treatment (5, 53, 54).

 Cell-based delivery systems are another approach, as demonstrated by Ling et al., who utilized engineered doxorubicin-loaded M1 macrophages (M1-Dox) to target cancer cells efficiently via a tunneling nanotube pathway. 

 This method exhibited higher efficiency in terms of effective concentration and drug loading than lysosomal delivery, proving effective in treating primary tumors and metastasis (55). Functional amyloids produced in bacteria, such as Pseudomonas exotoxin (PE24)-formed bacterial inclusion bodies, have shown promise. These functionalized inclusion bodies arrested tumor growth in colorectal cancer mouse models without toxicity (56). 

 Additionally, albumin, with multiple binding sites for cellular receptors and ligands, is a potential carrier for chemotherapy drugs targeting peritoneal metastasis, offering a biocompatible approach to drug delivery (5, 57).

 The treatment landscape for PC is multifaceted, incorporating surgical techniques, intraperitoneal chemotherapy, novel methods like PIPAC, and emerging approaches such as localized drug delivery systems and immunotherapies. While progress has been made, ongoing research and clinical trials are essential to refine and validate these treatment modalities, ultimately improving patient outcomes at different stages of PC.

***Impact OF FDG PET/CT on the treatment of PC***

 The utilization of ^18^F-FDG-PET/CT imaging has revolutionized the selection process for patients with PC who may benefit from cytoreductive surgery and HIPEC. This imaging modality combines the metabolic insight provided by FDG-PET with the anatomical detail of CT, offering a comprehensive overview of tumor activity and spread. Several studies have highlighted the crucial role of ^18^F-FDG PET/CT in accurately predicting intra-abdominal tumor load, which is essential for determining the feasibility of complete cytoreduction in PC cases (58–61). The Peritoneal Cancer Index (PCI) derived from PET/CT strongly correlates with surgical findings, guiding surgical planning and optimizing outcomes (58, 59). Combined PET/CT scans provide superior predictive value compared to separate PET and CT scans, offering detailed information on tumor extent and metabolic activity (58, 59). This imaging modality aids treatment planning by assessing tumor burden and spread within the peritoneal cavity, facilitating decisions on cytoreductive surgery and hyperthermic intraperitoneal chemotherapy (58–60). PET/CT imaging also assists in selecting appropriate candidates for these interventions, optimizing patient selection and treatment outcomes (58–60). 

 Additionally, research suggests PET/CT's superiority over multiphasic contrast-enhanced MRI in preoperative evaluations due to its higher diagnostic accuracy, better inter observer consistency, and stronger correlation with surgical outcomes (62).

 Differences in FDG PET/CT disease patterns can aid in better managing peritoneal carcinomatosis. 

 Diffuse Peritoneal Hyper metabolism:

 Signifying extensive disease involvement, it prompts consideration of palliative or aggressive therapies, reflecting advanced disease status. 

 Focal Peritoneal Hyper metabolism: Indicates discrete metastases or nodules, potentially leading to surgical resection or targeted therapies based on size, number, and distribution. Omental Caking: Characterized by thickened and hypermetabolic omentum, influencing treatment decisions such as systemic therapy, cytoreductive surgery, or HIPEC. 

Splenomegaly and Hepatomegaly: Reflecting metastatic involvement, guiding choices between systemic chemotherapy or targeted agents. Lymph Node Involvement: Abnormal suggests regional spread, influencing management options like surgery, radiation, or systemic therapy. Ascites and Peritoneal Thickening: impacting treatment planning for symptom relief and improving quality of life.

 The ability of ^18^F-FDG-PET/CT to detect small-volume disease and assess the response to therapy positions it as an indispensable tool in managing peritoneal carcinomatosis. By enabling a more targeted selection of patients for cytoreduction and HIPEC, this imaging technique ensures that the procedure's benefits are extended to those most likely to achieve a favourable prognosis while sparing others from an invasive procedure that may not offer survival advantage. The strategic integration of ^18^F-FDG-PET/CT in the treatment algorithm underscores its pivotal role in enhancing the precision of PC management.

## Conclusion

 In conclusion, integrating FDG PET/CT imaging has revolutionized PC management, offering precise insights into tumor burden, spread, and metabolic activity. By combining metabolic information from PET with anatomical detail from CT, this modality enhances patient selection for cytoreductive surgery and HIPEC, optimizing treatment outcomes. However, challenges persist, particularly in accurately characterizing lesions with diffuse involvement and intense metabolic activity. Despite these challenges, the strategic utilization of FDG PET/CT in the treatment algorithm underscores its pivotal role in enhancing the precision of PC management, ultimately improving patient care and prognosis. Continued research and advancements are necessary to overcome these challenges and further refine the utility of PET/CT imaging in PC management. The cases presented in this series give an insight into the clinical presentation, nature, pattern, and appearance of PC on FDG PET/CT. Moreover, it highlights the usefulness of FDG PET/CT in the process of clinical decision-making and the management of patients.
